# Immunotherapy for EGFR-mutant advanced non-small-cell lung cancer: Current status, possible mechanisms and application prospects

**DOI:** 10.3389/fimmu.2022.940288

**Published:** 2022-07-22

**Authors:** Chunyan Shi, Yan Wang, Jianxin Xue, Xiaojuan Zhou

**Affiliations:** ^1^ Department of Thoracic Oncology, Cancer Center, West China Hospital, Sichuan University, Chengdu, China; ^2^ The Department of Oncology, Jiujiang No.1 People’s Hospital, Jiujiang, China; ^3^ Department of Radiation Oncology, Cancer Center, West China Hospital, Sichuan University, Chengdu, China; ^4^ Laboratory of Clinical Cell Therapy, West China Hospital, Sichuan University, Chengdu, China

**Keywords:** EGFR mutation, immune checkpoint inhibitors, programmed cell death 1, programmed cell death ligand 1, tumor microenvironment, non-small-cell lung cancer

## Abstract

Immune checkpoint inhibitors (ICIs) are effective against advanced and even perioperative non-small-cell lung cancer (NSCLC) and result in durable clinical benefit, regardless of programmed death ligand-1 (PD-L1) expression status in cancer. Existing clinical evidence shows that the effect of immunotherapy in patients with EGFR-mutant NSCLC after the development of tyrosine kinase inhibitor (TKI) resistance is not satisfactory. However, compared with monotherapy, ICIs combined with chemotherapy can improve the efficacy. Encouragingly, compared with that of patients with sensitive mutations, the progression-free survival of patients with rare mutations who were treated with ICIs was increased. Adequately maximizing the efficacy of ICIs in EGFR-mutant NSCLC patients is worth exploring. In this review, we described preclinical and clinical studies of ICIs or combined therapy for EGFR-mutant NSCLC. We further focused on EGFR mutations and the cancer immune response, with particular attention given to the role of EGFR activation in the cancer-immunity cycle. The mechanisms for the natural resistance to ICIs were explored to identify corresponding countermeasures that made more EGFR-mutant NSCLC patients benefit from ICIs.

## Introduction

Among newly diagnosed patients with lung cancer, non-small-cell lung cancer (NSCLC) patients accounted for the highest proportion, approximately 80 percent of the total (1). In most countries, the 5-year survival rate of patients diagnosed with lung cancer between 2010 and 2014 was only 10% to 19% (2). In the past 20 years, many advances in molecular detection technology and molecular targeted therapy have shown promise for NSCLC patients. Epidermal growth factor receptor (EGFR) mutation is currently the most common target; approximately 10% to 15% of the Caucasian population and more than 50% of Asian patients with non-squamous cell carcinoma carry this mutation (3–5). Currently, clinical guidelines recommend EGFR-tyrosine kinase inhibitors (TKIs) as a first-line therapy for patients with advanced NSCLC (6–8) who are sensitive to EGFR mutations and do not harbor drug resistance genes. Compared with chemotherapy, treatment with first-generation and second-generation EGFR-TKIs has resulted in a median progression-free survival (PFS) of 9 to 13 months in patients with advanced NSCLC, and the median PFS provided by third-generation drugs was 18.9 months (9–12). However, nearly inevitably, patients acquire resistance within 9-19 months (13–15). Innovative therapies to overcome EGFR-TKIs resistance are still under investigation.

In research insights of the last few years, immune checkpoint inhibitors (ICIs), represented by programmed cell death receptor-1 (PD-1)/programmed death receptor ligand-1 (PD-L1) inhibitors, have attracted increasing attention due to durable clinical benefit along w1ith low toxicity in patients with NSCLC (16). Preclinical studies have shown that EGFR activation can upregulate the expression of endogenous PD-L1 on tumor cells, thus inducing apoptosis of T cells and promoting immune evasion of EGFR-mutant NSCLC (17). However, the fact that immunotherapy has little effect in advanced NSCLC patients with EGFR-sensitive mutations remains a challenge (18). Additionally, immunotherapy in these patients may be positively correlated with the development of hyperactive diseases, leading to increased toxicity and side effects (18, 19). However, in a phase I study of nivolumab (CheckMate 012), 21 patients with NSCLC harboring EGFR mutations received a combination of nivolumab and erlotinib, and the toxicity was tolerable (20).

Some reports have noted that tumor immunogenicity (21–23), the tumor microenvironment (TME) (24–26), copy number variations (27, 28), tumor-specific mutations, and specific intestinal bacteria (23) can influence the efficacy of ICIs. It was suggested that the low efficacy of ICIs in EGFR-mutant NSCLC was related to the specific TME, tumor mutation load (TMB) and PD-L1 expression level (29). The precise boundaries and interrelationships of these elements remain unclear and deserve further exploration. In this overview, we summarized recent studies on the application of PD-1/PD-L1 ICIs in EGFR-mutant NSCLC, mapped the cancer-immunity cycle of individual patients, and tried to explore the potential mechanisms leading to the poor clinical efficacy of ICIs in EGFR-mutant NSCLC, providing ideas for the development of specific immunotherapy or immunotherapy combinations.

## Clinical outcomes of ICIs for EGFR-mutant NSCLC patients

Recent clinical trials had found that ICI monotherapy has few effects on patients with EGFR mutations. However, in existing clinical studies, ICI combined with chemotherapy or anti-angiogenesis had achieved encouraging results. While ensuring the efficacy, the safety also should be guaranteed. Here, we reviewed the clinical efficacy and toxicity of ICIs in EGFR-mutant NSCLC (**Tables 1**–**3**).

**Table 1:** EGFR-mutant NSCLC patients benefit little from PD-1/PD-L1 monotherapy.

Clinical trial	Line	n	Treatment	ORR	Median PFS(months)	Median OS(months)	Safety	Phase
**CheckMate** **012** (30)	1	7	Nivolumab	14% in EGFRm, 30% in EGFRwt	1.8 in EGFRm *vs* 6.6 in EGFRwt	18.8 in EGFRm NR in EGFRwt	G3-4*: 1%, G5: 0%	1
**NCT02879994** (31)	1	11	Pembrolizumab	0%	–	–	TRAE: 46%, no G4-5	2
**NCT02008227/OAK** (32)	≥2	85	Atezolizumab (A) *vs* Docetaxel (D)	5%	NA	10.5 in EGFRm, 16.2 in EGFRwt;	G3-4:37%	3
**CheckMate 057** (33)	≥2	82	Nivolumab (N) *vs* docetaxel (D)	11%	HR1.46(0.90-2.37)	HR1.18(0.69-2.00);	G3-5*:10%	3
**KEYNOTE 010** (34)	≥2	86	Pembrolizumab (P) *vs* Docetaxel (D)	NA	HR1.79(0.94-3.42);	HR0.88(0.45-1.70)	G3-5*:13-16%	2/3
**POPLAR** (35)	≥2	19	Atezolizumab (A) *vs* docetaxel (D)	NA	NA	HR#: 0.99 in EGFRm *vs* 0.70 in EGFRwt with A *vs* D	G3-4*: 40%, G5: 4%	2
**KEYNOTE 001** (36)	≥2	74	Pembrolizumab *vs* docetaxel	4%	1.86	6.0 in EGFRm *vs* 11.9 in EGFRwt	G 3–5: 6%	1b
**PACIFIC** (37)	≥2	43	Durvalumab (D)	–	–	HR 0.76 (0.35~1.64)	G3-4: 29.9%	3
**ATLANTIC** (38)	≥3	102	Durvalumab -	3.6% for PD-L1 <25%, 12.2% for PD-L1≥25%	1.9	9.9 for PD-L1 <25%, 13.3 for PD-L1≥25%	G3-4: 5%	2
**WJOG8515L** (39)	≥2	102	Nivolumab (N) Carboplatin-pemetrexed (CP)	N *vs* CP: 9.6% *vs* 36.0%	N *vs* CP:1.7 *vs* 5.6	N *vs* CP:20.7 *vs* 19.9	TRAE:60.8% G3-5: 9.8%	2

NSCLC, non-small-cell lung cancer; EGFR, epidermal growth factor receptor; EGFRm, EGFR mutant; EGFRwt, EGFR wild type; PD-L1, programmed death-ligand 1; n, No. of EGFR mutant patients; ORR, overall response rate; OS, overall survival; PFS, progression-free survival; HR, hazard ratio; CI, confidence interval; TRAE, treatment related adverse event; AE, adverse event; G, grade of toxicity. *TRAEs for the entire study population and not selected for EGFRm patients. #OS or PFS data not given for EGFRm subgroup. NA, not applicable; TKIs, tyrosine kinase inhibitors.

**Table 2:** ICI-based immunotherapy combinations for EGFR-mutant NSCLC patients.

Clinical trial	Line	n	Treatment	ORR	Median PFS(months)	Median OS(months)	Safety	Phase
**with chemotherapy**								
**CheckMate 012** (40)	1	6	Nivolumab + PT-DC	17% in EGFRm *vs* 47% in EGFRwt	4.8in EGFRm *vs* 7.5 in EGFRwt	20.5 in EGFRm *vs* 24.5 in EGFRwt	TRAE:7%, G3-4*:50%, Pneumonitis most common	1
**IMpower 130** (41)	≥2	NA	Atezolizumab + PT-DC *vs* PT-DC	NA	7.0 *vs*. 6.0 HR, 0.75	14.4 *vs*. 10, HR = 0.98;	G3-5*: 32% *vs* 28%	3
**NCT03513666** (42)	≥2	40	Toripalimab + PT-DC	50%	7.0	23.5	TRAE:97.5%, G3 -5: 65.0%	2
**with CTLA-4 blockade**								
**CheckMate 012** (43)	1	8	Nivolumab + ipilimumab -	50%	–	–	TRAE*: 72-82%, G3-4: 33-37%, no G5	1
**KEYNOTE 021** (44)	≥2	10	Pembrolizumab + Ipilimumab	10% in EGFRm *vs* 30%EGFRwt	-	-	TRAE*:98%, G3-G5: 49%, one G5 pancreatitis	1/2
**ICIs + VEGF Inhibitor + Chemotherapy**								
**IMpower150** (45)	≥2	34	Atezolizumab (A) + bevacizumab (B) + carboplatin-paclitaxel (CP) Atezolizumab (A) + carboplatin-paclitaxel (CP); bevacizumab (B) + carboplatin-paclitaxel (CP)	70.6% for ABCP, 35.6% for ACP, 41.9% for BCP	10.2 for ABCP, 6.1 for BCP HR 0.38(0.21–0.68)	NR for ABCP, 17.5 for BCP	G3-4: 64% of ABCP, 68% of ACP, and 64% of BCP	3
**NCT03647956** (46)	≥2	40	Atezolizumab + bevacizumab + pemetrexed-carboplatin	62.5%	9.43	the 1-year OS rate was 72.5%.	G3-5: 37.5%, One G5 myocardial infarction	2

NSCLC, non-small-cell lung cancer; EGFR, epidermal growth factor receptor; EGFRm, EGFR mutant; EGFRwt, EGFR wild type; PD-L1, programmed death-ligand 1; n, No. of EGFR mutant patients; ORR, overall response rate; OS, overall survival; PFS, progression-free survival; HR, hazard ratio; CI, confidence interval; TRAE, treatment related adverse event; AE, adverse event; G, grade of toxicity. *TRAEs for the entire study population and not selected for EGFRm patients. NA, not applicable; NR, not reached; TKIs, tyrosine kinase inhibitors; PT-DC, platinum-doublet chemotherapy; ICIs, immune checkpoint inhibitors.

**Table 3:** ICIs combined with EGFR-TKIs enhanced EGFR-mutant NSCLC patient toxicity.

Clinical trial	Line	n	Treatment	ORR	Median PFS(months)	Median OS(months)	Safety	Phase
**NCT02088112** (47)	1	56	Gefitinib + durvalumab dose escalation	63.3%-70%	10.1-12.0	-	TRAE: 100%, G3-5 hepatotoxic AEs:42.5%	1
**NCT02013219** (48)	1	20	Atezolizumab + erlotinib	75%	15.4	32.7	G3: 43% no G4-5 occurred.	1b
**KEYNOTE 021 cohort E and F** (49)	1	19	Pembrolizumab + erlotinib(n=12)/ gefitinib (n=7)	Erlotinib 41.7%, gefitinib 14.3%	Erlotinib19.5,gefitinib1.4	Erlotinib NR, Gefitinib 13.0	P+E: TRAE: 100%, G3:33.3%, no G4-5 P+G: TRAE: 85.7%, G3-4 hepatotoxic AEs: 71.4%	1/2
**Myung-Ju Ahn, et al.** (50)	1	11	Durvalumab + Osimertinib	82%	9.0	terminated early owing to ILD	35%ILD	1b
**Myung-Ju Ahn, et al.** (50)	≥2	23	Durvalumab + Osimertinib	43%	DOR:20.4	-	50% diarrhea, 41% nausea, 35% appetite decreased	1b
**TATTON** (51)	≥2	23	Durvalumab + Osimertinib	43%	–	–	TRAE:100%, G3-5: 48%. 22% ILD with 8.7% G≥3	1b
**CAURAL** (52)	≥2	14	Durvalumab + Osimertinib *vs* Osimertinib	64%	NR *vs*19.3	NR	TRAE:100%, G3-5: 8%. One G2 ILD reported	3
**CheckMate 012** (53)	≥2	21	Nivolumab + erlotinib	15%	5.1	18.7	G3: 24%, no G4-G5	3

NSCLC, non-small-cell lung cancer; EGFR, epidermal growth factor receptor; EGFRm, EGFR mutant; EGFRwt, EGFR wild type; PD-L1, programmed death-ligand 1; n, No. of EGFR mutant patients; ORR, overall response rate; OS, overall survival; PFS, progression-free survival; DOR, duration of response; HR, hazard ratio; CI, confidence interval; TRAE, treatment related adverse event; AE, adverse event; G, grade of toxicity. *TRAEs for the entire study population and not selected for EGFRm patients. NR, not reached; TKIs, tyrosine kinase inhibitors; ICD, interstitial lung disease; ICIs, immune checkpoint inhibitors.

### PD-1/PD-L1 monotherapy

The phase III clinical trial CheckMate 057 confirmed (33) that patients with advanced NSCLC who were treated with nivolumab survived longer than those treated with docetaxel during or after platinum-based chemotherapy, and it was reported for the first time that ICIs did not improve PFS or overall survival (OS) in NSCLC patients with EGFR mutations. Meta-analysis data from three clinical trials (CheckMate 057, POPLAR and KEYNOTE 010) proved that PD-1/PD-L1 ICIs did not prolong OS (HR=1.05, 95% CI: 0.70-1.55, P=0.81) in EGFR-mutant NSCLC patients (35) compared with docetaxel. In addition, a meta-analysis of five trials (CheckMate 017, CheckMate 057, KEYNOTE 010, OAK and POPLAR) reported by Lee et al. also confirmed that prolonged OS was not observed in the EGFR mutations subgroup (16). Most studies have shown that PD-1 monotherapy may be ineffective in patients with EGFR-mutant NSCLC (**Table 1**).

### ICI-based immunotherapy combinations

Conventional chemotherapeutic drugs can promote recovery of immune surveillance function in tumor patients. Therefore, it was hypothesized that the ideal clinical effect can be obtained by adding ICIs to chemotherapy in patients with NSCLC with EGFR mutations. In the first-line setting of CheckMate 012, PFS and OS were 4.8 and 20.5 months, respectively, in the EGFR-mutant group, while PFS and OS were 7.5 and 24.5 months, respectively, in the EGFR wild-type group that received combination therapy of nivolumab and chemotherapy (**Table 2**) (40). In another phase II study of NCT03513666, when receiving a combination of toripalimab and chemotherapy, the objective response rate (ORR) was 50%, and the median PFS was 7 months for EGFR-mutant NSCLC after TKI resistance (42). The effects of the combination of chemotherapy and ICIs are ambiguous because of the small sample size, and more large clinical studies are worth further exploration.

In addition to its well-known antiangiogenic effect, bevacizumab has also been found to mediate immune regulation (54–56). The results of an open-label phase III study, Impower 150 (NCT02366143), seemed to confirm this hypothesis. Regardless of PD-L1 expression and gene alterations in patients with metastatic NSCLC who did not receive chemotherapy, the PFS (8.3 months *vs*. 6.8 months) and OS (19.2 months *vs*. 14.7 months) of patients in the chemotherapy ± atezolizumab + bevacizumab group (ABCP group) were significantly longer than those of patients in the chemotherapy + bevacizumab group (BCP group) (57). Unfortunately, in patients with EGFR mutations, OS did not benefit in the ABCP group compared with the BCP group (HR=0.61, 95% CI 0.29-1.28) (57). ORIENT-31 was the first phase III study to confirm that ICI combined with antiangiogenic therapy and chemotherapy significantly improved PFS in EGFR-mutant non-squamous NSCLC patients with EGFR-TKIs treatment progress (58). The PFS was prolonged in group A (sintilimab + IBI305 + chemotherapy) compared with group C (placebo1 + placebo2 + chemotherapy): 6.9 months *vs*. 4.3months (HR=0.750, 95% CI 0.337-0.639; P<0.0001). And the confirmed ORR were 43.9% and 25.2% in group A and group C respectively. The combination of ICI and antiangiogenic therapy creates a new pattern of EGFR-TKIs resistance.

In theory, cytotoxic T-lymphocyte antigen-4 (CTLA-4) and PD-1 have a coordinated effect on antitumor immune responses. The combination of ipilimumab and nivolumab was used as the first-line treatment for EGFR-mutant NSCLC, and the ORR was 50% (43). In a subgroup of phase II study KEYNOTE 021, when receiving a combination of ipilimumab and pembrolizumab, the ORR was 10% for EGFR-mutant NSCLC after TKIs resistance and 30% for the EGFR wild-type group. These trials indicated that the efficacy of double ICIs needs further confirmation (44).

### The heterogeneity of EGFR-mutant subtypes

**Figure 1:**
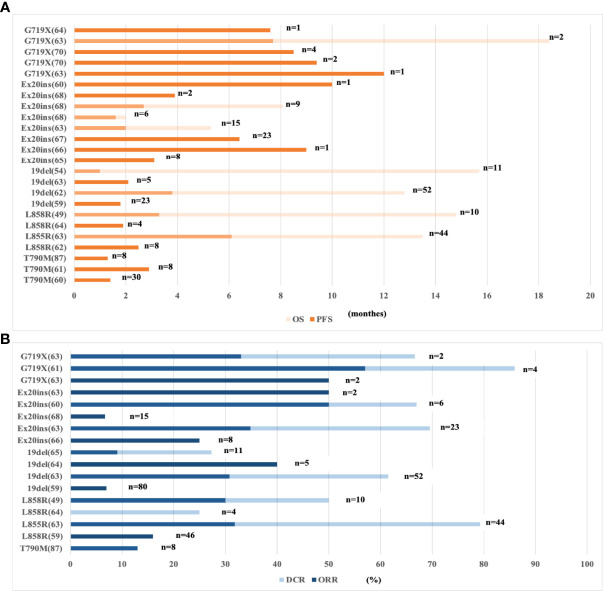
Clinical data of ICI-based immunotherapy for subtypes of EGFR-mutant NSCLC patients. **(A)** The PFS and OS of ICI-based immunotherapy for subtypes of EGFR-mutant NSCLC. **(B)** The ORR and DCR of ICI-based immunotherapy for subtypes of EGFR-mutant NSCLC. NSCLC, non-small-cell lung cancer; EGFR, epidermal growth factor receptor; n, No. of EGFR mutant patients; ORR, overall response rate; DCR, disease control rate; OS, overall survival; PFS, progression-free survival; ICIs, immune checkpoint inhibitors; 19del, exon 19 deletion; Ex20 ins, exon 20 insertion.

In a multicenter retrospective clinical cancer study, 171 NSCLC patients with EGFR mutations were treated with PD-1/PD-L1 ICIs or ICIs combined with a CTLA4 inhibitor (59). Immunotherapy was less effective in patients with EGFR exon 19 deletion or L858R mutation than in patients with wild-type EGFR. In addition, the efficacy in the exon 19 deletion group was worse than that in the exon L858R group (ORR, 22% in the wild-type subgroup, 16% in the L858R subgroup, and 7% in the EGFR exon 19 deletion subgroup). New evidence from several recent studies suggested that NSCLC patients with rare EGFR mutations had a priority response to ICIs (**Figure 1**). Chen et al. showed that the good response of patients with rare EGFR mutations in NSCLC, including patients with exon 20 insertion or G719X, L861Q, or S768I mutations (69), was associated with the concomitant expression of PD-L1 in the TME (70) and the high incidence of CD8^+^ tumor-infiltrating lymphocytes (TILs). The heterogeneity of the TME of distinct EGFR mutations results in different immune responses to ICIs. Further exploration of the pathological and immunological characteristics of different subtypes may help us select the population benefiting from ICIs.

### The toxicity of ICIs

From existing preclinical and clinical studies, ICIs were generally well tolerated as monotherapy or in combination with chemotherapy or anti-angiogenesis for EGFR-mutant NSCLC patients, and no newly treatment-related adverse events have been observed. However, it is worth noting that ICIs combined with EGFR-TKIs enhanced toxicity among EGFR-mutant NSCLC patients.

Some studies have shown that EGFR-TKIs can induce the immunogenic apoptosis of tumor cells, recruit T cells or upregulate the expression of PD1/PD-L1. Therefore, it is logical to combine TKIs with ICIs. Dismally, in clinical trials, the combined application of EGFR-TKIs and ICIs for the treatment of EGFR-mutant NSCLC patients did not provide a significant clinical benefit but increased side effects (**Table 3**). Compared with previous studies of EGFR-TKIs monotherapy, pembrolizumab combined with erlotinib did not improve ORR in the phase I/II KEYNOTE-021 trial (NCT02039674) (49). Moreover, five of the seven patients treated with pembrolizumab combined with gefitinib developed grade 4 hepatotoxicity, which led to premature termination of treatment. In clinical, the application of ICIs should be cautiously considered in patients receiving EGFR-TKIs.

## Probable mechanisms responsible for ICI resistance in EGFR-mutant NSCLC

**Figure 2:**
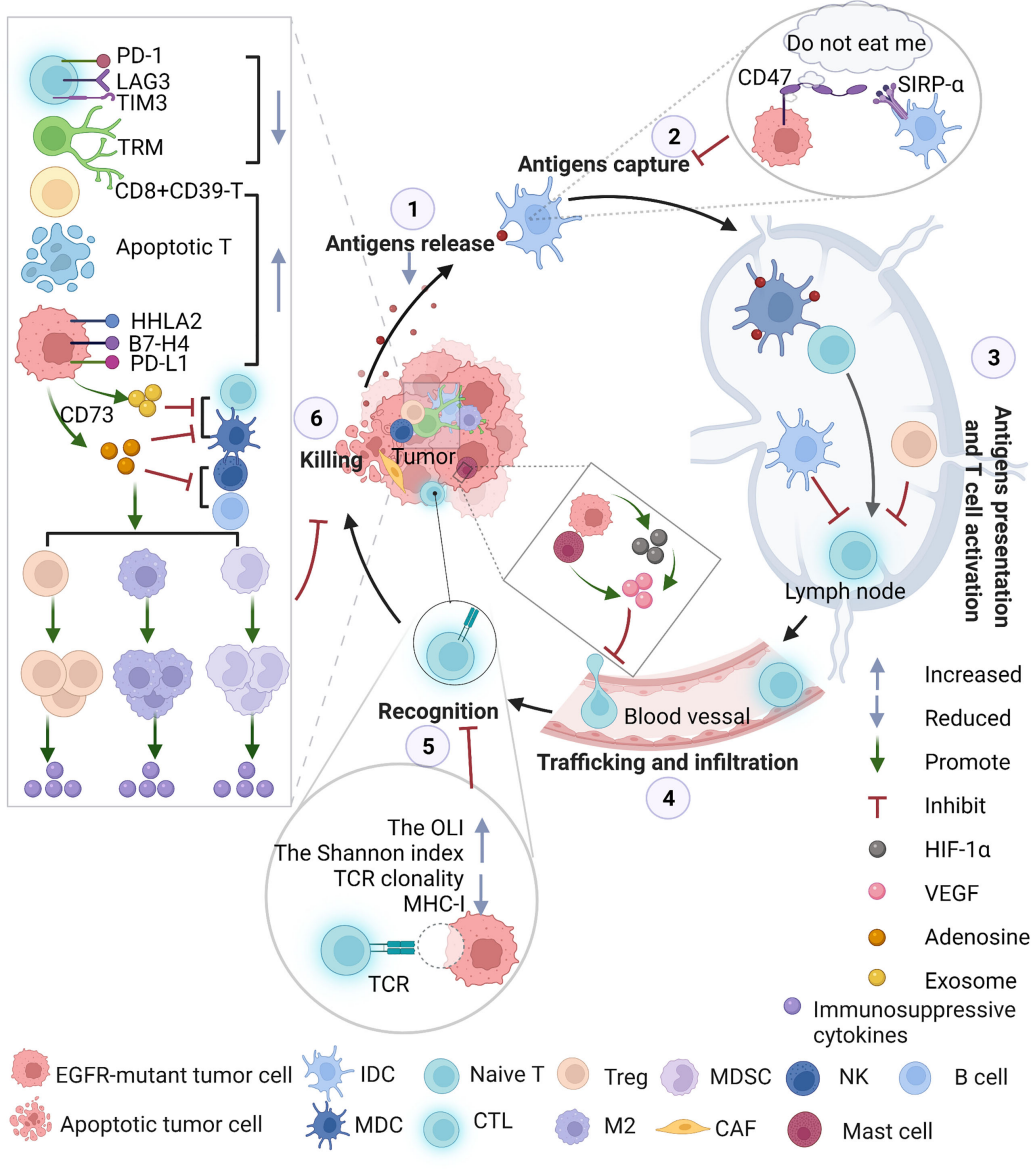
The immunosuppressive TME throughout the whole cancer-immunity cycle in EGFR-mutant NSCLC. EGFR activation alters immune profiles through the following pathways: the surface of cancer cells creates a “do not eat me” signal that inhibits professional phagocytic cells, such as dendritic cells (DCs), from engulfing cancer cells due to the presentation of tumor antigens; promotes CTLA-4 expression to enhance the inhibitory function of Tregs; increases the infiltration of Tregs in the TME and promotes tumor growth; increases mast cells that contribute to angiogenesis and induces neovascularization by releasing proangiogenic factors; decreases CD8^+^ T-mediated antitumor activity, inhibiting the expression of MHC (**Figure 2**); enhances T-cell apoptosis, promoting the M2-like polarization of macrophages and increasing the levels of IL-10, CCL13, GDF15, CCL23, CXCL17, TGF-β, soluble PD-L1 and CCL2. CCL2 plays a critical role in the migration of MDSCs to the TME. MDSCs exert antitumor immunosuppressive actions, such as producing immunosuppressive molecules, inhibiting antitumor functions, inducing T-cell apoptosis, and upregulating Tregs. CAFs, with characteristics of MDSCs, in EGFR-mutant NSCLC might interfere with the immune response. EGFR-mutant tumors secrete exosomes containing EGFR mutations or PD-L1 to promote distant metastasis. EGFR-mutant tumor cells may change metabolic pathways, such as upregulating CD73 and converting ATP to adenosine. Massive adenosine exerts immunosuppressive activity on a variety of immune cells: Tregs and accumulation of MDSCs, further attenuating antitumor function in NKs, B cells and DCs activity, skews Mφ polarization toward M2 macrophages and inhibits the CTL-mediated antitumor response, mediating tumor immune evasion. NKs, natural killer cells; DCs, dendritic cells; IDC, immature dendritic cells; MDC, mature dendritic cells; Tregs, Treg cells; MHC, major histocompatibility complex; MDSC, myeloid-derived suppressor cells; EGFR, epidermal growth factor receptor; TME, tumor microenvironment; ATP, adenosine triphosphate; PD-L1, programmed death-ligand 1; Mφ, macrophages; CTL, cytotoxic T lymphocytes.

Collectively, according to preclinical and clinical trials, EGFR-mutant NSCLC patients benefit little from ICIs, especially ICI monotherapy, due to their heterogeneous immune characteristics. Encouragingly, compared with that of patients with sensitive mutations, the PFS of patients with rare mutations who were treated with ICIs was increased. At present, the specific mechanism that causes this phenomenon is not clear and is worth exploring further. Daniel S. Chen et al. (71) proposed that by understanding the individualized biological features of patients, biomarkers associated with tumor immunity may enable us to track the cancer-immunity cycle of specific patients and customize precision immunotherapy or combinatorial immunotherapy. In the following paragraphs, we describe the main steps in the characteristic cancer-immunity cycle of patients with EGFR-mutant NSCLC (**Figure 2**) to explore the potential mechanism underlying the poor immunotherapy response.

### Decreased release of MHC-I and MHC-II neoantigens

The T-cell-mediated anticancer response starts with the release of new antigens produced by tumorigenesis that are captured by antigen presenting cells (APCs). Immunogenic cell death (ICD) accompanied by the release of neoantigens is an irritation signal; tolerable or apoptotic cell death is an inhibitory signal (72). Wu et al. (73) reported that the specific T-cell response to the clonal tumor antigen encoded by EGFR-driven mutation was successfully identified in a patient with advanced EGFR-mutant NSCLC who benefited from ICIs after developing TKIs resistance. In other words, due to the presence of new, highly immunogenic, and specific clonal antigens, ICIs have potential application in NSCLC patients with acquired drug resistance to EGFR-TKIs. Unfortunately, somatic mutations and predicted major histocompatibility complex (MHC) class I and class II neoantigens were significantly lower in EGFR-mutant NSCLC than in EGFR wild-type tumors (P < 0.01) (74), which inhibited anticancer responses and promoted immune evasion. From this point of view, the investigation of ICD will provide new approaches for tumor treatment in EGFR-mutant NSCLC. Practically, a study has proven that combined antigen-capturing treatment and ICIs have a positive impact on the cancer-immunity cycle (75).

### Decreased ability to capture cancer antigens

Recently, an *in vitro* study by Nigro et al. (76) showed that gefitinib-induced downregulation of CD47 expression can promote phagocytosis of cancer cells by reactive cells, while the establishment of gefitinib resistance can reverse this response. When exposed to increasing drug concentrations, the expression of CD47 (a “do not eat me” signal) on the surface of PC9GR cells that were resistant to gefitinib was significantly increased. Blocking the CD47/SIRPα axis by adding a CD47-specific monoclonal antibody can significantly increase the phagocytosis of PC9GR by dendritic cells (DCs). Similarly, an *in vivo* experiment confirmed that administration of CD47-specific monoclonal antibodies significantly inhibited the growth of lung cancer patient-derived xenotransplant tumors *via* recruitment of macrophages into the TME (77).

### Restriction of cancer antigen presentation and T-cell activation

A recent study *in vivo* showed that EGFR E746-A750 deletion mutant lung cancer can induce DC anergy and inhibit antitumor immunity, while T-cell infiltration and DC function were restored; meanwhile, the efficacy of ICIs in EGFR-19del tumors was improved because of the use of TKIs in combination with granulocyte-macrophage colony-stimulating factor (78). Moreover, DCs in tumors show different phenotypes and draining lymph nodes, and a marked reduction in the proliferative activity of T cells in lymph nodes was also observed.

Regardless of whether tumor antigens are captured and presented internally by APCs or transmitted externally, T-cell activation is another strategy for interfering with the cancer-immunity cycle. In addition to homologous antigen recognition, costimulatory signals are needed for optimal T-cell activation, while in tumor tissues, not only the levels of costimulatory ligands but also the levels of MHC molecules are reduced by immunosuppressive factors (79). Some researchers analyzed the TME in mice with EGFR-driven tumors: among TILs, the ratio of CD8^+^/CD4^+^ T cells to CD8^+^/Foxp3^+^ T cells was markedly decreased compared with that in normal lung tissue (80). In detail, they found a significant increase in PD-1^+^ and Foxp3^+^ T cells in tumors, and PD-1 was expressed on most Foxp3^+^ T cells. In other words, the PD-1 pathway and Tregs are the main factors that inhibit the function of effector T cells. It is conceivable that blocking PD-1 in the EGFR-driven mouse model of lung cancer did not change the number of Tregs expressing high levels of CTLA-4, while combined dual ICIs may have a coordinated effect.

### Inhibition of T cells trafficking and infiltration into tumors

**Figure 3:**
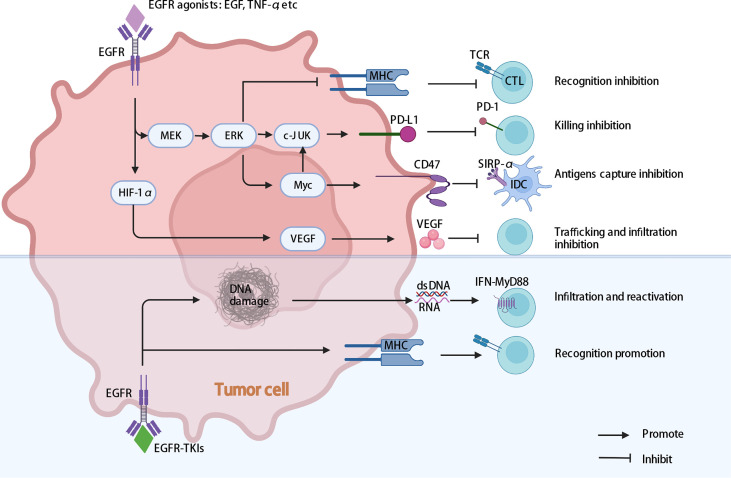
Multiple intrinsic cancer cell pathways induce cancer cell immune evasion in EGFR-mutant NSCLC. EGFR activating mutations may help cancer cells escape cytotoxic T-cell recognition and specific killing by promoting PD-L1 expression and downregulating MHC expression. The activation of EGFR may influence the expression of VEGF, inhibiting T lymphocyte infiltration into tumors, generating vascular endothelial growth and promoting tumor progression. In addition, activation of EGFR may influence the expression of CD47, decreasing the phagocytosis of cancer cells by DCs. In addition, EGFR-TKIs enhance MHC expression, and HypoTKI can induce more dsDNA and RNA release and trigger MyD88–type I IFN innate sensing pathways, which enhance tumor-specific T-cell infiltration and reactivation. EGFR, epidermal growth factor receptor; MEK/ERK, extracellular signal-regulated kinase (ERK) kinase MEK; MHC, major histocompatibility complex; EGFR-TKIs, epidermal growth factor receptor tyrosine kinase inhibitors; HypoTKI, low-fractionated EGFR-TKIs; DCs, dendritic cells.

Under the action of cell adhesion molecules and chemokine receptors, activated T cells leave lymph nodes, enter the blood, roll along the endothelium, exude from the blood circulatory system and either infiltrate into or surround the tumor mass (81, 82). In EGFR-mutant NSCLC, EGFR signaling plays an important role in tumor invasion activity by regulating hypoxia-independent hypoxia inducible factor-1α (HIF-1α) and vascular endothelial growth factor (VEGF) expression. Cells with acquired resistance to EGFR-TKIs maintain high levels of HIF-1α and VEGF expression, and this pathway is no longer regulated by EGFR (83). VEGF expression interferes with the infiltration of CD8^+^ T cells into tumor tissue (84), while angiogenesis and tumor growth continue (**Figure 3**).

Early studies confirmed that EGFR signaling activates c-Jun/c-Jun N-terminal kinase and reduces interferon regulatory factor-1 expression; the former increases CCL22 expression to recruit CD4^+^ regulatory T cells, while the latter reduces the induction of CD8^+^ T-cell infiltration *via* CXCL10 and CCL5 (85). Unfortunately, a number of clinical studies on the combination of standard-dose TKIs and ICIs have been stopped prematurely due to severe side effects. Further investigation suggested that low-fractionated EGFR-TKIs (HypoTKI) were more effective than standard hyperfractionated EGFR-TKIs (HyperTKI) because HypoTKI can induce more dsDNA and RNA release than HyperTKI *in vivo* and trigger MyD88–type I IFN innate sensing pathways, which enhance tumor-specific T-cell infiltration and reactivation. More importantly, blocking with ICIs had a synergistic effect without serious side effects (86). In conclusion, therapies that improve T-cell transport and infiltration may act synergistically with ICIs in EGFR-mutant NSCLC.

### Decreased ability of T cells to recognize tumors

Antigens captured by MHC-I and MHC-II molecules are presented to T cells by APCs. Some studies have shown that an activating mutation in EGFR suppresses the expression of MHC-I in NSCLC through the extracellular signal-regulated kinase (ERK) kinase MEK pathway, which leads to the poor response of NSCLC to immunotherapy. Homoplastically, other studies suggested that in patients with EGFR-TKI resistance, T790 M-negative tumors tended to respond more favorably to the ICI nivolumab than T790 M-positive cells (87). In T790 M-positive tumors, the activation of the EGFR pathway remains unchanged, which may lead to inhibition of MHC-I expression. These results suggest that EGFR-TKIs combined with ICIs can improve the response to immunotherapy. However, data from several early studies conducted simultaneously with EGFR-TKIs and ICIs in patients with NSCLC were disappointing (88–91), showing high toxicity due to adverse events, such as interstitial lung disease and elevated liver enzymes (89, 90). Therefore, further investigation to determine the best treatment strategy for the simultaneous or continuous use of EGFR-TKIs or MEK inhibitors and ICIs in EGFR-mutant NSCLC is needed.

The T-cell receptor (TCR) lineage consists of thousands of TCR clones, reflecting an individual’s immunity during aging, infection and even malignancy. It is of high clinical value to distinguish the clonality and diversity of TCR (Shannon index, richness, etc.) and the overlap index (OLI) of unique TCR chain sequences identified between tissue and blood. One study found that obvious curative effects of ICIs can be seen in patients who had high clonality and high OLI scores (92–94). The NADIM clinical trial NCT03081689T identified two parameters from TCR sequence analysis as predictive biomarkers of complete pathologic response (CPR) after neoadjuvant chemoimmunotherapy, which may be superior to the PD-L1 tumor proportional score (TPS) and TMB, and revealed the possible mechanism by which CPR is involved in enhancing tumor immunogenicity and peripheral immune monitoring (95). Researchers analyzed a total of 39 pairs of normal and tumor lung tissue samples (20 cases with EGFR mutations), and the TCR diversity index was found to be significantly elevated, while the clonal expansion of T cells in EGFR mutant tumors was compared with that in EGFR wild-type tumors. In whole exon group sequencing, the nonsynonymous mutations and predicted new antigen expression levels were markedly decreased in EGFR mutant tumors (96). Similarly, other researchers collected and studied samples from 93 patients with NSCLC and divided them according to EGFR mutation and subtype (94). They found that the different responses to ICIs in patients were attributed to the presence of differences in TCR clonality, the Shannon index and the OLI of different EGFR subtypes. These findings may partly explain the molecular mechanism underlying the poor response to ICIs in patients with EGFR mutations.

### Remodeled ability of T cells to kill tumor cells

TILs are the most critical cell group infiltrating tumor nests and stroma. The higher the density of CD8^+^ TILs is, the better the immune effect (97, 98). An increasing number of studies have revealed that EGFR-mutant NSCLC cells alter the TME to limit TILs and suppress T-cell-mediated immune attack (99). Zhao et al. discussed the mechanism underlying the low abundance of tumor-infiltrating CD8^+^ T cells in EGFR-mutant NSCLC: the exosomes secreted by the EGFR-mutant NSCLC lines PC9 and HCC827 promoted the apoptosis of CD8^+^ T cells more than the EGFR wild-type cell lines H1299 and SK-MES-1 (100). In addition, a retrospective study suggested that tumors with rare EGFR mutations benefited more from ICIs that were rich in TILs (101). Similarly, in a retrospective analysis of 58 patients who received ICIs after EGFR-TKIs, correlation analysis showed a significant negative correlation between TKI-PFS and the corresponding IO-PFS (102). Furthermore, the proportion of TILs in patients with short TKI-PFS was higher, and the ratio of M2-like macrophages to M1-like macrophages was lower. Moreover, Simoni et al. (103) showed that a large number of bystander CD39^-^CD8^+^ T cells in EGFR-mutated tumor cells led to poor reactions to ICIs, while the proportion of CD39^+^ CD8^+^ TILs was visibly higher in patients with wild-type EGFR. In addition, coinhibitory molecules, such as PD-L1, PD-1, TIM-3, TIGIT, and LAG-3, play essential and fundamental roles in immune suppression (104). Notably, HHLA2, a newly discovered member of the B7/CD28 family, contributed to tumor immunosuppression by regulating T-cell function and was not detected in most normal lung tissues, but an expression rate of 66% was observed in different subtypes of NSCLC (105). In particular, compared with that in wild-type NSCLC, the expression of HHLA2 in EGFR-mutant NSCLC was relatively high. HHLA2 may become a new target in the exploration of strategies to improve the efficacy of ICIs in EGFR-mutant NSCLC.

Immunosuppressive cells recruited by EGFR-mutant NSCLC cells can negatively regulate the killing ability of T cells. Wang et al. (106) confirmed for the first time that the EGFR signaling pathway was closely related to Tregs regulation. EGFR signal activation causes more Tregs to be generated and activated (107, 108). Gefitinib reduced the inhibition of EGFR signaling in the TME by decreasing Treg numbers in tumors (109).

The metabolic pathway of tumor cells and related products affects the immune killing function of T cells. The single-cell transcriptome indicated that EGFR-mutant NSCLC cells had more genes related to metabolic pathways, which was crucial for the negative impact on the TME (110). The adenosine signaling axis was thought to have a wide range of immunosuppressive effects on the TME, including inhibiting the lytic activity of cytotoxic T lymphocytes (CTLs) and natural killer cells (NKs) and enhancing the proliferation of Tregs, myeloid-derived suppressor cells (MDSCs) and inhibitory macrophages (111, 112). CD73 is a critical enzyme in the conversion of AMP to adenosine and an exo-50-nucleotidase encoded by the NT5E gene (113). It was found that the expression of CD73 in EGFR-mutant NSCLC was significantly increased compared with that in EGFR wild-type cell lines (114). CD73 blockade markedly inhibited tumor growth in a mouse model of EGFR-mutant NSCLC. It seems to be understood that CD73 may cause EGFR mutations in NSCLC with a low response rate to ICIs, but Ishii et al. (115) revealed that in patients with EGFR-mutant NSCLC, high CD73 expression showed greater protective ICI effects. The role of the CD73 adenosine pathway in EGFR-mutated NSCLC needs to be validated in more experiments.

From what has been discussed above, current studies on tumor immunotherapy have mainly focused on T-cell immunity, and inhibitory factors exist in every link of the cancer-immunity cycle, which seems to explain the poor efficacy of ICIs for EGFR-mutant NSCLC patients. From this perspective, it was necessary to combine with other treatment strategies to break these adverse conditions and to make EGFR-mutant NSCLC patients benefit from ICIs.

## Potential strategies to improve efficacy of immunotherapy in EGFR-mutant NSCLC

In general, EGFR-mutated NSCLC responds poorly to ICI monotherapy, but some subgroups may benefit, especially in combination with chemotherapy and/or antiangiogenic agents. In view of the characteristics of the different responses of ICIs in EGFR-mutant subgroups, individualized diagnosis and treatment measures need to be formulated in clinical practice. Here, we scientifically envisioned several promising strategies to improve ICI efficacy in EGFR-mutant NSCLC after TKI resistance.

### Combined chemotherapy and/or anti-angiogenesis treatment

Considering the improved benefits of NCT03513666A and the IMpower150 trial, a promising treatment was to combine ICIs with chemotherapy to improve the immunogenicity of tumor cells or anti-angiogenesis to promote more TIL infiltration into the tumor in EGFR-mutant NSCLC. Further optimization schemes and more are under way, such as the CheckMate-722, ABC-lung and NCT04147351 studies.

### Novel ICIs

According to T-cell-targeting immunomodulator immunology, another promising treatment to overcome the poor efficiency of ICIs is to target other ICIs associated with the TME (116). Several clinical studies against novel ICIs, such as LAG3, TIGIT, and B7-H3, are ongoing for NSCLC. Zhou reported that LAG-3 was upregulated after TKI resistance in EGFR-mutant NSCLC (71), which provided novel insights for the anti-LAG treatment of EGFR-mutant NSCLC patients. The exploration of more novel ICIs in monotherapy or combination therapy may provide more and better treatment options for EGFR-mutated NSCLC after TKI resistance.

### Combined radiotherapy

Radiotherapy causes random point mutations and double-strand breaks in DNA, increases the effects of TMB and new antigens, and can provide good local tumor control, thus playing an important role in the treatment of lung cancer (117). Radiotherapy can lead to ICD and the release of high migration group box 1 protein (HMGB-1); HMGB-1 binds to Toll-like receptor-4 (TLR-4), participates in the progression and presentation of tumor antigens, and promotes the activation and maturation of DCs. Through a series of the abovementioned pathways, the immunogenicity of tumor cells is enhanced (118–121). Radiotherapy also increases the expression of natural killer group 2 member D (N-K-G2-D) and the first apoptotic signal and promotes the recognition and clearance of tumor cells by T cells and NKs (122, 123). In short, radiotherapy can transform noninflammatory tumors (also known as “cold” tumors) into inflammatory tumors (also known as “hot” tumors) through complex mechanisms, increase tumor immunogenicity and increase sensitivity to ICIs.

A retrospective analysis of the KEYNOTE 001 study showed that the good prognosis resulting from ICIs was closely related to having received radiotherapy (124). Another prospective clinical study of high-dose fractionated radiotherapy combined with ICIs, PEMBRO-RT, confirmed that radiotherapy improved the ORR of ICIs (125). For patients with EGFR-sensitive mutated NSCLC, a number of clinical studies have confirmed that consolidation radiotherapy during EGFR-TKIs significantly prolongs PFS and total OS (126, 127). Of course, the secondary T790 M mutation in patients with EGFR-TKI resistance will also affect the OS of patients, while Ouyang et al. found that whether patients received radiotherapy before developing drug resistance did not affect the occurrence of acquired T790 M mutation (128). It is suggested that radiotherapy can not only reduce tumor load and prolong the time until acquisition of drug resistance to TKIs but also prolong the total survival of patients through its immunomodulatory effect.

In summary, future exploration should focus on verifying whether radiotherapy can effectively change the TME in EGFR-mutant NSCLC and which radiotherapy can maximally activate immunity.

### Cancer vaccines

Vaccination can accelerate anticancer immunity by inhibiting negative regulatory factors (129). In one study (130), an EGFRT790 M/C797S mutant-derived peptide (MQLMPFGSLL) that can bind to human leukocyte antigen (HLA)-human leukocyte antigen was identified, and an EGFRT790 M/C797S- peptide-specific CTL clone isolated from human PBMCs from healthy HLA-A2 donors showed high responsiveness to cancer cells because T2 cells pulsed with the EGFRT790 M/C797S peptide suffered strong cytotoxicity. Immunotherapy targeting new antigens that arise from EGFR mutations or in combination with ICIs may be a useful new therapeutic strategy for patients who are resistant to osimertinib.

In a recent major trial (131), 24 patients with grade III/IV NSCLC who developed progressive disease after a variety of conventional treatments, including surgery, radiotherapy, chemotherapy and TKI therapy, received a personalized neoantigen peptide vaccination (PPV). Immunosurveillance showed that five of the seven patients with EGFR-mutant NSCLC showed a vaccine-induced T-cell response to EGFR neoantigen peptide. All of these patients showed an increase in the frequency of neoantigen-specific CD8^+^ T cells in peripheral blood after PPV. These results suggest that personalized neoantigen vaccination is a viable, safe and well-tolerated option for patients with advanced NSCLC. The neoantigen peptide displayed by human leukocyte antigen molecules on the surface of tumor cells shows exquisite tumor specificity and can cause T-cell-mediated tumor rejection. However, it is predicted that there are few neoantigens shared among patients; therefore, more preclinical and clinical data on vaccination are needed.

### Bypass vaccination through adoptive T-cell therapy

Eshhar et al. proposed for the first time that chimeric antigen receptor (CAR)-targeted T-cell therapy was a promising strategy for the treatment of malignancies (132). Han’s team (133) infused an increasing dose of EGFR-targeted CAR-T cells into patients with EGFR positivity (> 50% expression) and recurrent/refractory NSCLC in a phase I clinical study (NCT01869166). Of the 11 assessable patients, 2 achieved partial remission, and five were stable for 2 to 8 months. The infusion of EGFR-targeted CAR-T cells was safe and well tolerated and resulted in no severe toxicity. The pathological clearance of EGFR-positive tumor cells after treatment and detection of the CAR-EGFR gene in tumor-infiltrating T cells in all four patients were observed in tumor biopsies. EGFR targeting CAR-T cells are a safe and feasible option for the treatment of advanced EGFR-mutant NSCLC. At present, current research is still focused on EGFR-positive lung cancer, but CAR-T-cell treatment of EGFR-mutant NSCLC also needs to be further studied. Of course, potential efficacy assessments and safety assessments have not been fully conducted, including whether the transfer of a large number of monospecific T cells will lead to drug resistance due to antigenic drift and whether the identified toxicity problems can be safely addressed.

### Target B cells and related products

Increasing evidence has proven that the significant efficacy of B cells may promote both the response and prognosis of ICIs (134). Compared with the EGFR-wild-type group, the proportion of plasma cells was lower in the EGFR-mutant NSCLC group (110). A more recent study established that the disappearance of follicular helper CD4^+^ T (TFH)-B-tissue-resident memory CD8^+^ T (TRM) cooperation mediated by the CXCL13-CXCR5 axis in EGFR-mutant NSCLC may account for poor responses to ICIs (135). Patient-derived antibodies are involved in the regulation of the TME (136); therefore, it was worthwhile to further explore the roles of B cells in EGFR-mutant NSCLC.

## Conclusion

In summary, achieving the goal of complete and safe cancer eradication through ICIs may require only monotherapy in a few patients with EGFR-mutant NSCLC, while most of these patients may need combination therapy, and the major challenge for the latter group is joint toxicity. In addition, current studies suggested that EGFR L858R, a common mutation, and rare mutation still showed superiority in ICIs treatment. More preclinical and clinical studies exploring the combination of ICIs and several other treatments, such as anti-angiogenesis, chemotherapy, novel ICIs, radiotherapy, anti-CD47-SIRP-α, anti-CD73- adenosine axis, and B-cell-associated immunity are urgently needed because most analyses are based on subgroup analysis or retrospective studies. Moreover, the optimal dose, sequence and schedule of the combination should also be included in future studies. However, in basic research, it was challenging to obtain humanized animal models containing EGFR mutations, which seriously restricts the progress of research. We speculated that continued improvement of the mouse preclinical model would accelerate the pace of ICI optimization in EGFR-mutant NSCLC patients.

Through the cancer-immunity cycle, immunotherapy has informed promising approaches for EGFR-mutant advanced NSCLC patients. Considering the large population of EGFR-mutant NSCLC patients and low toxicity and durable clinical benefit of ICIs, it is particularly important to explore immunotherapy strategies after TKI resistance.

## Author contributions

CS wrote the original draft. YW collected and interpreted the data. CS and YW analyzed data, prepared, and reviewed figures. JX offered significant guidance. XZ offered main direction of this manuscript. CS and YW contributed equally to this work. All authors contributed to the article and approved the submitted version.

## Conflict of interest

The authors declare that the research was conducted in the absence of any commercial or financial relationships that could be construed as a potential conflict of interest.

## Publisher’s note

All claims expressed in this article are solely those of the authors and do not necessarily represent those of their affiliated organizations, or those of the publisher, the editors and the reviewers. Any product that may be evaluated in this article, or claim that may be made by its manufacturer, is not guaranteed or endorsed by the publisher.
